# The Statistical Scalpel: Sharpening Clinical Research Through Statistical Literacy in Neurosurgery: A Short Review: Short title: A Statistical Guide for Neurosurgery Research

**DOI:** 10.31661/gmj.v15i.4142

**Published:** 2026-06-01

**Authors:** Ehsan Jangholi, Parivash Hafez Amini, Neda Pak, Atieh Hosseinkhani, Kamkar Aeinfar, Seyed Mohammad Ghodsi, Milad Shafizadeh, Mohammadreza Boustani

**Affiliations:** ^1^ Department of Neurosurgery, Shariati Hospital, Tehran University of Medical Sciences, Tehran, Iran; ^2^ Brain and Spinal Cord Injury Research Center, Neuroscience Institute, Tehran University of Medical Sciences, Tehran, Iran; ^3^ Department of Physiology, Faculty of Medicine, Istanbul Atlas University, Istanbul, Turkey; ^4^ Department of Radiology, Children Medical Center of Excellence, Tehran University of Medical Sciences, Tehran, Iran; ^5^ Neurosurgical Intensive Care Unit, Shariati Hospital, Tehran University of Medical Sciences, Tehran, Iran; ^6^ Department of Neurosurgery, Aja University of Medical Science, Tehran, Iran

**Keywords:** Biostatistics, Neurosurgery, Research Methodology, Evidence-Based Medicine, Statistical Tests

## Abstract

Modern neurosurgery clearly relies on evidence-based medicine. The integrity, interpretation, and application of research depended on a solid understanding of the statistical methods used. This review highlights the important need for neurosurgeons and trainees to gain proficiency in basic statistical concepts. We emphasize this need throughout the research process—from forming hypotheses and defining variables to selecting tests, analyzing power, and interpreting key indices. Also, we provide practical guides for choosing suitable statistical tests and translating research findings into useful clinical metrics. We believe that statistical literacy is not an extra skill but a key part of clinical expertise and academic success. It allows neurosurgeons to evaluate literature, conduct valid research, and effectively apply evidence in patient care.

## Introduction

In neurosurgery, where decisions markedly impact patient survival and quality of life, relying on solid scientific evidence is crucial. While technical skills are developed in the operating room, the ability to critically assess and create scientific evidence comes from understanding research. A key part of this understanding is knowing biostatistics. For neurosurgeons, statistics are not just abstract numbers; they are essential tools that change raw data into clinical insights. This helps distinguish between real advances and misleading connections [1].

For instance, when evaluating a new endoscopic technique for the removal of pituitary adenoma, a surgeon might find a study that claims a statistically significant reduction in operating time. However, suppose they do not check the confidence interval (CI) for the mean difference (MD; e.g., -5 to 25 minutes). In that case, they may overlook that the clinical significance is questionable, as the interval includes both negligible and potentially important reductions [2].

Hence, this article aims to provide a comprehensive and accessible framework as a short educational review, applying core statistical principles throughout the research process.

## The Research Lifecycle: Statistical Impact

### Formulating the Research Question and Study Design

Every phase of a clinical research project is closely linked to statistical thinking. A well-defined research question is the foundation. Statistical considerations directly shape how this question is framed and investigated. For example, a spine surgeons group wants to compare a new minimally invasive spine fusion technique versus a conventional open approach. Here, the choice of primary outcome (e.g., “fusion rate at 6 months” vs. “mean change in Oswestry Disability Index score”) determines the statistical tests. The study design randomized controlled trial (RCT), cohort, or case-control affects the strength of causal inference and susceptibility to bias. Understanding confounding variables (e.g., patient age, bone density) is also vital at this stage to design a study that can control for them [3].

Understanding Variables: The Building Blocks of Data The type of data collected determines the statistical methods used. Variables are broadly classified as: A. Categorical Variables These include groups or categories and have two subtypes, as nominal those that have no true order (e.g., tumor type: glioma, meningioma, metastasis; surgical approach: anterior, posterior), and ordinal, which categorizes with a meaningful order, but the intervals between them cannot be measured (e.g., ASA physical status classification, GCS score, McCormick scale for spinal cord function). B. Continuous Variables These are measurable quantities that represent an interval or ratio. In other words, they have a true zero point, allowing for statements like “twice as much” (e.g., bleeding volume in mm, tumor size in cm, intracranial pressure [ICP] in mmHg). For example, if comparing post-operative pain between two craniotomy techniques using a verbal rating scale (none, mild, moderate, severe), the data is ordinal. Using a t-test (suited for continuous data) would be incorrect; the Mann-Whitney U test is the right choice [4].

Sample Size Calculation and Power: A Priori Imperative A study with insufficient participants is unclear and might even be unethical; therefore, knowing about statistical power is crucial. Sample size calculation relies on four key parameters: significance level (α, typically 0.05), power (1-β, typically 80-90%), the minimum clinically important effect size, and an estimate of the standard deviation [5]. Hence, we could input these parameters into statistical software (like PASS, G*Power) or online sample size calculators. The software will determine the minimum number needed for each group based on the type of study design (e.g., comparing two means, two proportions) and the parameters. A study with low power might miss a real effect (Type II error), while an overpowered study might find statistically significant but clinically irrelevant differences. For example, a study with low power on a new aneurysm clipping technique might not detect a real decrease in recurrence risk, potentially leading to dismissing a better method. In contrast, a study with too much power might find a statistically significant difference in operating time that is not clinically meaningful. In another example, if a new drug is believed to reduce vasospasm after a subarachnoid hemorrhage by an absolute 10%, calculating the sample size is vital to ensuring the trial has a high chance of finding this effect if it exists [5].

### The Special Case of Retrospective Studies and Post-hoc Power

Most of the initial research by trainees involves retrospective analyses of existing data. Here, the sample size is fixed (e.g., all patients over five years, N=450). Therefore, the question shifts from “How many subjects do I need?” to “What is the power of my study with the available sample?” Accordingly, performing a post-hoc power analysis is crucial for confirming a study’s findings, especially negative results. Suppose a study with a specific sample size has low power (<80%) to detect a clinically relevant effect. In that case, a non-significant result (p>0.05) cannot be trusted as evidence of “no difference.” So, reporting this analysis shows methodological rigor and prevents misleading conclusions from low-powered retrospective studies [6]. In another example, assume a retrospective review shows no significant difference in infection rates between two skull fixation techniques (P=0.47). However, a post-hoc analysis might reveal that the study had only 30% power to detect a 5% absolute difference. The conclusion should be “the study was inconclusive” rather than “no difference was found.”

### Data Analysis and Interpretation of Results

Misinterpretation at this point can lead to incorrect clinical conclusions. The three most important key concepts include: P-value: A P-value less than 0.05 suggests the observed effect is unlikely to be due to chance alone; it does not measure the probability that the research hypothesis is true [7]. CI: A 95% CI for an MD that does not cross zero (e.g., 2.1 to 5.3) is equivalent to a p-value < 0.05. More importantly, the width of the CI indicates how precise the estimate is—a narrow CI means greater precision [2]. Clinical vs. Statistical significance: A result can be statistically significant but without any clinical importance [8]. Example: A study finds that a new neuroprotective drug reduces mean ICP by 1 mmHg with a P=0.04. While this is statistically significant, a 1 mmHg reduction is unable to change patient management. The statistical finding is valid, but the clinical impact is minimal.

## Selecting the Right Tool: A Guide to Common Statistical Tests

**Table:** Table1. Guide to Selecting Common Statistical Tests for Neurosurgical Research

Research Purpose	Variable Type	Recommended Statistical Test
Compare two independent groups	Continuous, normally distributed outcome	Student’s t-test (e.g., mean blood loss between two craniotomy approaches)
	Continuous, non-normal or ordinal outcome	Mann-Whitney U test (e.g., median hospital stay with/without post-op CSF leak)
Compare 3+ groups	Continuous, normally distributed outcome	ANOVA (e.g., mean extent of resection across glioma subtypes)
	Continuous, non-normal or ordinal outcome	Kruskal-Wallis test (e.g., median improvement in ASIA score across spinal injury protocols)
Examine relationship	Two continuous variables	Pearson’s Correlation (for linear relationships, e.g., age vs. blood loss)
	Two continuous or ordinal variables	Spearman’s Rank Correlation (for monotonic relationships, e.g., surgeon experience vs. operative time)
Examine association	Two categorical variables	Chi-square test (e.g., association between antiplatelet use and post-op hemorrhage)
Predict an outcome	Multiple predictors vs. an ordinal outcome	Ordinal Logistic Regression (e.g., predict GOS score after TBI based on age, GCS, etc.)
	Multiple predictors vs. time-to-event outcome	Cox Proportional Hazards Regression (e.g., predict survival after GBM resection based on MGMT status, EOR, etc.)

**Table:** Table2. Essential Statistical Indices for the Neurosurgeon-Researcher

Statistical Indexes	Definition and Clinical Interpretation	Study Type Source(s)
RR	Ratio of risk in intervention vs. control. e.g., RR=0.33 means a 67% reduction in relative risk.	RCTs, Cohort
ARR	Absolute difference in risk. e.g., ARR=8% means the intervention absolutely reduces risk by 8 percentage points.	RCTs, Cohort
OR	Ratio of odds in exposed vs. control.e.g., OR=2.5 means the odds of the outcome are 2.5 times higher in the exposed group.	Case-Control, Cohort
HR	Instantaneous risk ratio over time.e.g., HR=0.7 means a 30% lower rate of the event (e.g., death) in the treatment group at any time point.	Survival studies
NNT	Patients needed to be treated to prevent one adverse outcome, which is calculated as 1/ARR. e.g., NNT=50 means 50 patients must be treated to prevent one event.	Derived from RCTs (via ARR)
Sensitivity	Proportion of true positives correctly identified.	Diagnostic test accuracy survey
Specificity	Proportion of true negatives correctly identified.	
Type I Error (α)	False positive (concluding an effect exists when it does not).	All interventional studies
Type II Error (β)	False negative (failing to detect a real effect).	
MD	Difference in means of a continuous outcome between two groups.e.g., MD = -150 mL provides a tangible measure of a treatment’s benefit (e.g., reduced blood loss).	RCTs, Cohort

**RR:** Risk Ratio or Relative Risk; **ARR:** Absolute Risk Reduction; **OR:** Odds Ratio; **HR:** Hazard Ratio; **NNT:** Number Needed to Treat; **RCT:** Randomized Controlled Trial; **MD:** Mean Difference

The choice of the right statistical test is critical and depends on the research question as well as the type of variables involved. Table1 could offer a helpful guide for making this selection.

Key factors include whether the data meet assumptions of normality (for parametric tests) and how many groups are being compared. Hence, before checking the table, ask yourself three questions:

1. What is the purpose of the analysis? (comparison group(s), examine relationships or associations)

2. What is the type of dependent variable (outcome)? What is the main outcome of your study? Is it quantitative or qualitative?

3. What is the independent variable (predictor), and how many groups does it have? What is the comparison factor?

## Interpreting the Evidence: Key Statistical Indices for Clinical Decision-Making

Moving beyond p-value to understand the impact as well as the clinical relevance of an effect is essential. In Table2, important statistical indices help translate research findings into practical clinical insights.

## Application at the Bedside: From Journal to Patient

The final mission of research is how it influences patient care. A neurosurgeon who understands statistics can effectively bridge this gap.

### Informed Consent

When talking about a new surgical intervention, the surgeon can clarify the evidence by using ARR and NNT. For example, “a technique lowers your risk of revision surgery from 10% to 5%, meaning we need to perform it on 20 patients to avoid one revision (NNT=20).” Hence, it is more informative than providing only a relative risk reduction [9]. Critical Appraisal When reviewing an article, a surgeon who understands bias and confounding can critically assess whether the positive results apply to their own patient population [10].

### Multidisciplinary Communication

Discussing outcomes at a tumor board requires precision. Stating, “the progression-free survival was significantly longer, with a median increase of 4 months and a HR of 0.7,” delivers a clear, statistically based, and clinically relevant message.

## The Role of Neurosurgical (NS) Trainees and Faculty

For residents and fellows, statistical proficiency is a core competency required for designing research, passing academic evaluations, and critically consuming the literature. Faculty should incorporate statistical reasoning into educational conferences, journal clubs, and research supervision [11, 12].

## Common Statistical Pitfalls in NS Research

The NS research presents unique methodological challenges that, if not properly addressed, can lead to significant statistical pitfalls and misleading conclusions. Moving beyond basic instruction, it is crucial to recognize these field-specific issues:

### Small Sample Sizes and Low Event Rates

The rarity of many NS pathologies often leads to studies with limited participants and few outcome events. This inherently reduces statistical power and increases the risk of Type II errors (failing to identify a true effect). In such contexts, a non-significant p-value (P> 0.05) is particularly uninformative. Researchers should prioritize reporting effect sizes with CIs to convey the range of plausible effects. For very small samples or rare events, exact statistical tests (e.g., Fisher’s exact test) or Bayesian methods may be more appropriate than traditional asymptotic methods [13].

### Misinterpretation of p-values and Neglect of Effect Sizes

A sole reliance on p-values, without reporting CIs and clinically meaningful effect sizes (e.g., MD, ARR), remains common and can be misleading [2, 7].

### Inadequate Control of Confounding

In observational studies, factors like disease severity, surgeon experience, and patient comorbidities can confound results. Multivariable regression or propensity score matching are essential to address this [3].

### Misapplication of Statistical Tests

Using parametric tests (e.g., t-test) for non-normally distributed or ordinal data (e.g., surgical rating scales) is a frequent error. Non-parametric alternatives should be considered [4].

### Barriers to Biostatistical Collaboration

Despite recognizing the importance of sound statistics, many NS units face practical hurdles in implementing robust biostatistical collaboration. These barriers can include limited funding for statistician co-investigators, a lack of integrated statistical support within departments, and a cultural gap between clinical and methodological experts. To overcome this, proactive efforts are needed: seeking statisticians as integral members of the research team from the project’s inception, utilizing institutional clinical and translational science institutes, and fostering a collaborative environment where methodological questions are valued as highly as clinical ones [14]. Investing in this collaboration is not a luxury but a necessity for generating high-impact, valid NS evidence.

## Conclusion

Statistical literacy is not an ancillary skill but a fundamental component of modern NS expertise. It is central to training, enabling neurosurgeons to critically evaluate literature, conduct valid research, and make informed patient-care decisions.

Investing in robust statistical education and mentorship is imperative; it directly translates into higher-quality research, reduces methodological pitfalls, and ultimately leads to safer, more effective, and evidence-based patient outcomes.

For the future of neurosurgery, sharpening our statistical scalpels is as crucial as honing our surgical ones.

## Conflict of Interest

The authors have no competing interests to declare that are relevant to the content of this article.

## AI Disclosure Statement

During the preparation of this manuscript, the authors used liboberry.com for language editing, grammar improvement. After its use, the authors thoroughly reviewed, verified, and revised all AI-assisted content to ensure accuracy and originality. The authors take full responsibility for the integrity and final content of the published article.
